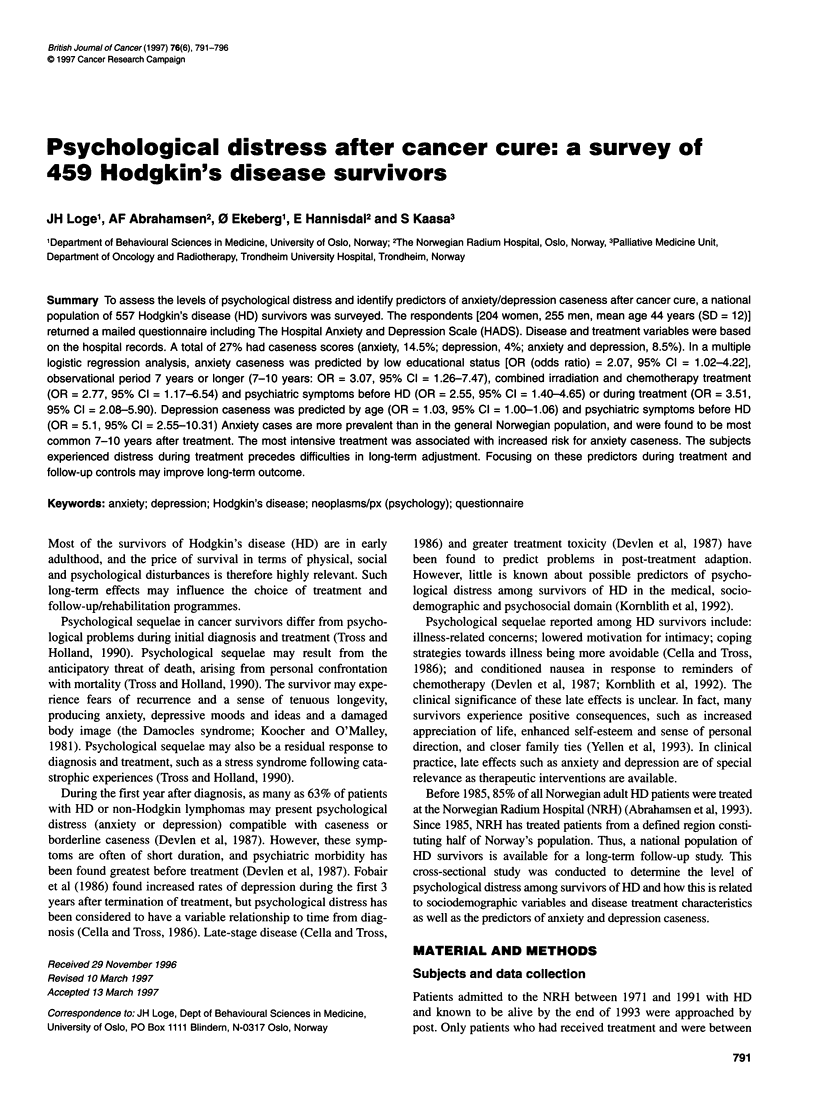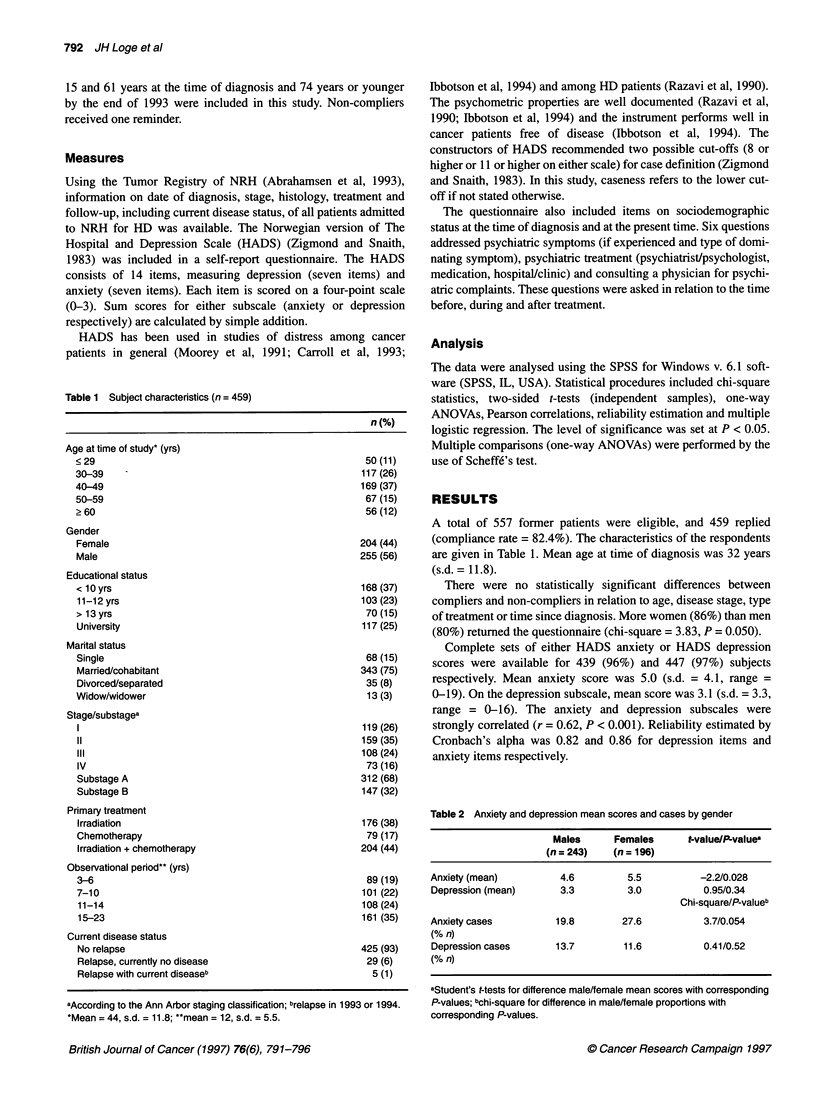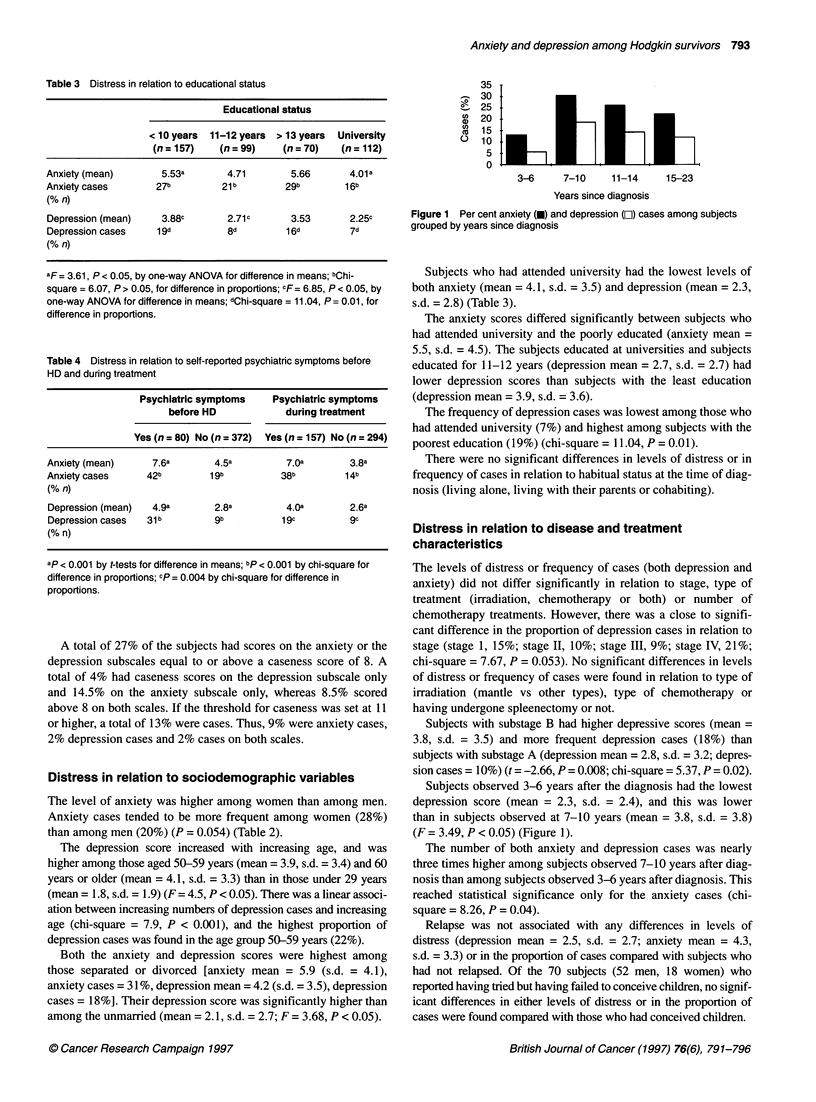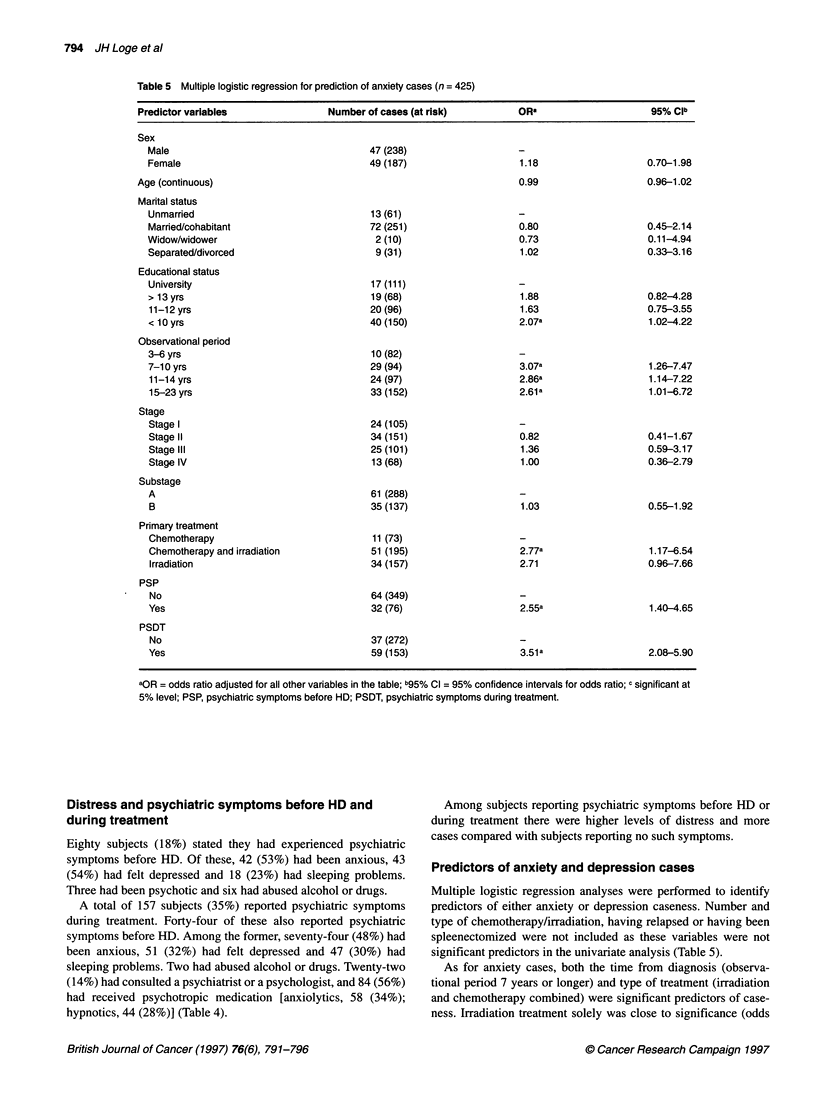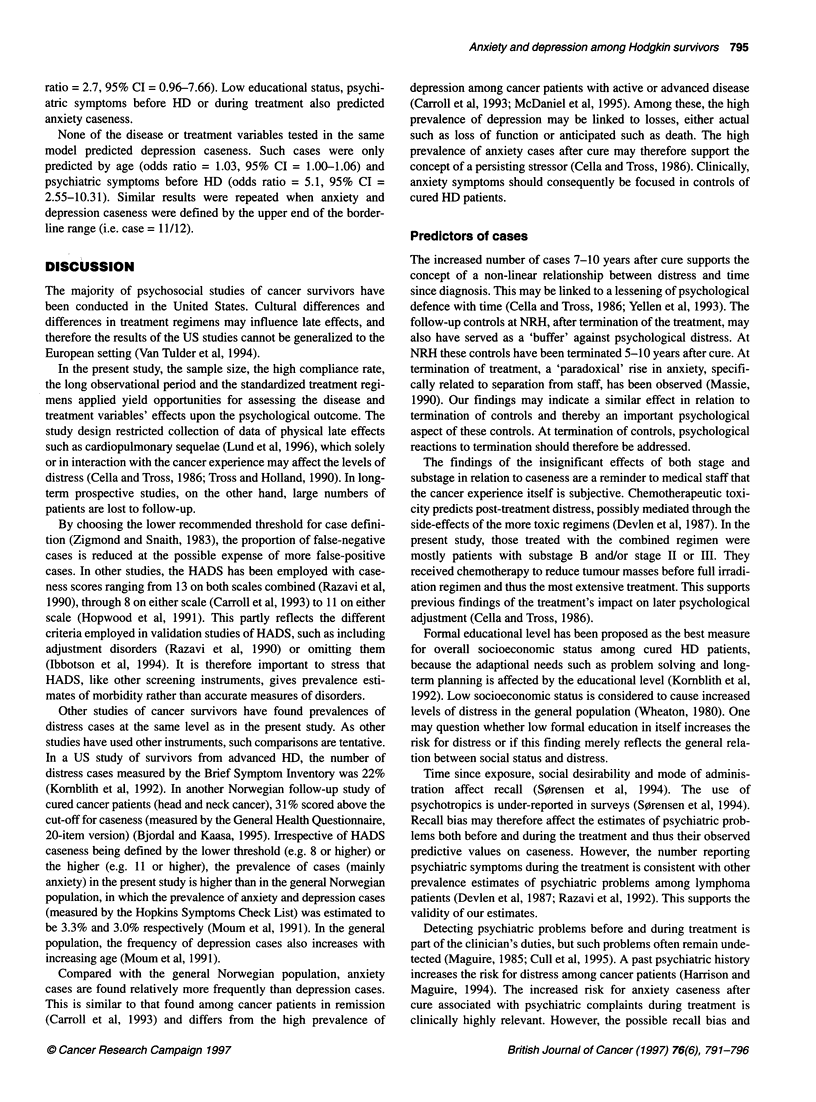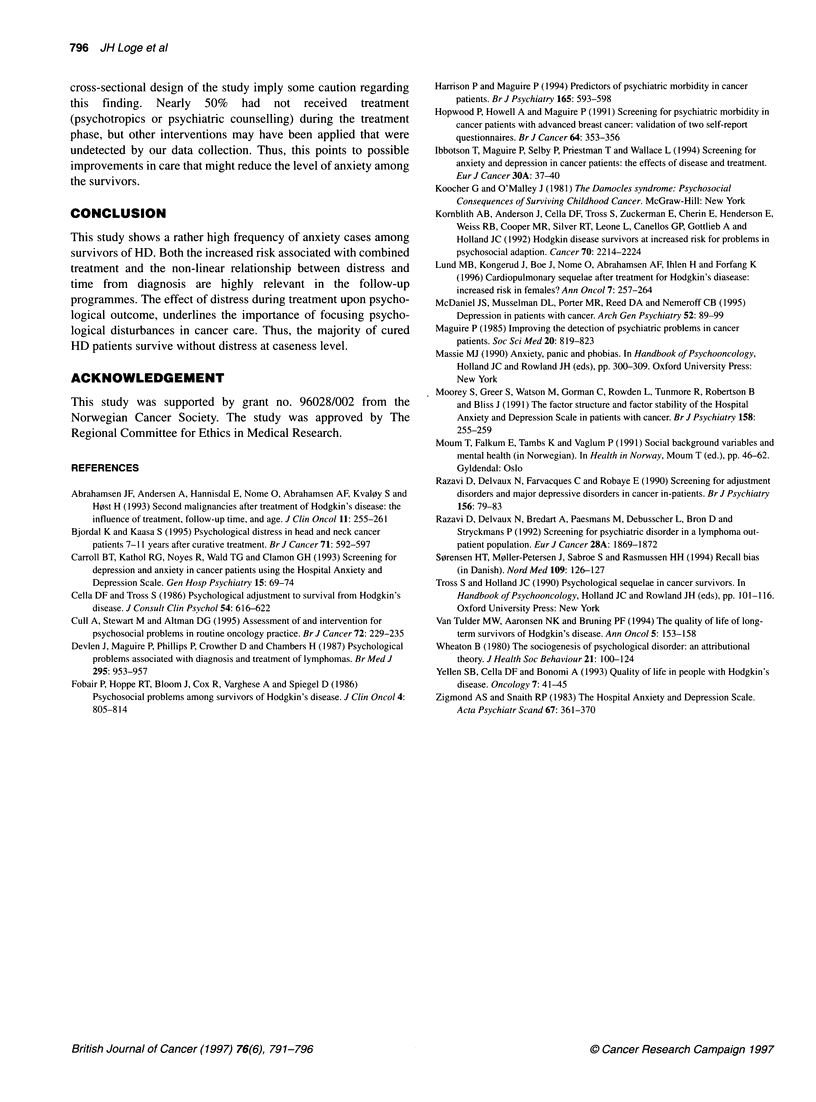# Psychological distress after cancer cure: a survey of 459 Hodgkin's disease survivors.

**DOI:** 10.1038/bjc.1997.464

**Published:** 1997

**Authors:** J. H. Loge, A. F. Abrahamsen, O. Ekeberg, E. Hannisdal, S. Kaasa

**Affiliations:** Department of Behavioural Sciences in Medicine, University of Oslo, Norway.

## Abstract

To assess the levels of psychological distress and identify predictors of anxiety/depression caseness after cancer cure, a national population of 557 Hodgkin's disease (HD) survivors was surveyed. The respondents [204 women, 255 men, mean age 44 years (SD = 12)] returned a mailed questionnaire including The Hospital Anxiety and Depression Scale (HADS). Disease and treatment variables were based on the hospital records. A total of 27% had caseness scores (anxiety, 14.5%; depression, 4%; anxiety and depression, 8.5%). In a multiple logistic regression analysis, anxiety caseness was predicted by low educational status [OR (odds ratio) = 2.07, 95% CI = 1.02-4.22], observational period 7 years or longer (7-10 years: OR = 3.07, 95% CI = 1.26-7.47), combined irradiation and chemotherapy treatment (OR = 2.77, 95% CI = 1.17-6.54) and psychiatric symptoms before HD (OR = 2.55, 95% CI = 1.40-4.65) or during treatment (OR = 3.51, 95% CI = 2.08-5.90). Depression caseness was predicted by age (OR = 1.03, 95% CI = 1.00-1.06) and psychiatric symptoms before HD (OR = 5.1, 95% CI = 2.55-10.31) Anxiety cases are more prevalent than in the general Norwegian population, and were found to be most common 7-10 years after treatment. The most intensive treatment was associated with increased risk for anxiety caseness. The subjects experienced distress during treatment precedes difficulties in long-term adjustment. Focusing on these predictors during treatment and follow-up controls may improve long-term outcome.


British Journal of Cancer (1997) 76(6), 791–796
© 1997 Cancer Research Campaign

### Psychological distress after cancer cure: a survey of 459 Hodgkin's disease survivors

**JH Loge[1], AF Abrahamsen[2], Ø Ekeberg[1], E Hannisdal[2] and S Kaasa[3]**

[1]Department of Behavioural Sciences in Medicine, University of Oslo, Norway; [2]The Norwegian Radium Hospital, Oslo, Norway, [3]Palliative Medicine Unit, Department of Oncology and Radiotherapy, Trondheim University Hospital, Trondheim, Norway

**Summary** To assess the levels of psychological distress and identify predictors of anxiety/depression caseness after cancer cure, a national population of 557 Hodgkin's disease (HD) survivors was surveyed. The respondents [204 women, 255 men, mean age 44 years (SD = 12)] returned a mailed questionnaire including The Hospital Anxiety and Depression Scale (HADS). Disease and treatment variables were based on the hospital records. A total of 27% had caseness scores (anxiety, 14.5%; depression, 4%; anxiety and depression, 8.5%). In a multiple logistic regression analysis, anxiety caseness was predicted by low educational status [OR (odds ratio) = 2.07, 95% CI = 1.02–4.22], observational period 7 years or longer (7–10 years: OR = 3.07, 95% CI = 1.26–7.47), combined irradiation and chemotherapy treatment (OR = 2.77, 95% CI = 1.17–6.54) and psychiatric symptoms before HD (OR = 2.55, 95% CI = 1.40–4.65) or during treatment (OR = 3.51, 95% CI = 2.08–5.90). Depression caseness was predicted by age (OR = 1.03, 95% CI = 1.00–1.06) and psychiatric symptoms before HD (OR = 5.1, 95% CI = 2.55–10.31) Anxiety cases are more prevalent than in the general Norwegian population, and were found to be most common 7–10 years after treatment. The most intensive treatment was associated with increased risk for anxiety caseness. The subjects experienced distress during treatment precedes difficulties in long-term adjustment. Focusing on these predictors during treatment and follow-up controls may improve long-term outcome.

**Keywords:** anxiety; depression; Hodgkin's disease; neoplasms/px (psychology); questionnaire

Most of the survivors of Hodgkin's disease (HD) are in early adulthood, and the price of survival in terms of physical, social and psychological disturbances is therefore highly relevant. Such long-term effects may influence the choice of treatment and follow-up/rehabilitation programmes.

Psychological sequelae in cancer survivors differ from psychological problems during initial diagnosis and treatment (Tross and Holland, 1990). Psychological sequelae may result from the anticipatory threat of death, arising from personal confrontation with mortality (Tross and Holland, 1990). The survivor may experience fears of recurrence and a sense of tenuous longevity, producing anxiety, depressive moods and ideas and a damaged body image (the Damocles syndrome; Koocher and O'Malley, 1981). Psychological sequelae may also be a residual response to diagnosis and treatment, such as a stress syndrome following catastrophic experiences (Tross and Holland, 1990).

During the first year after diagnosis, as many as 63% of patients with HD or non-Hodgkin lymphomas may present psychological distress (anxiety or depression) compatible with caseness or borderline caseness (Devlen et al, 1987). However, these symptoms are often of short duration, and psychiatric morbidity has been found greatest before treatment (Devlen et al, 1987). Fobair et al (1986) found increased rates of depression during the first 3 years after termination of treatment, but psychological distress has been considered to have a variable relationship to time from diagnosis (Cella and Tross, 1986). Late-stage disease (Cella and Tross, 1986) and greater treatment toxicity (Devlen et al, 1987) have been found to predict problems in post-treatment adaption. However, little is known about possible predictors of psychological distress among survivors of HD in the medical, sociodemographic and psychosocial domain (Kornblith et al, 1992).

Psychological sequelae reported among HD survivors include: illness-related concerns; lowered motivation for intimacy; coping strategies towards illness being more avoidable (Cella and Tross, 1986); and conditioned nausea in response to reminders of chemotherapy (Devlen et al, 1987; Kornblith et al, 1992). The clinical significance of these late effects is unclear. In fact, many survivors experience positive consequences, such as increased appreciation of life, enhanced self-esteem and sense of personal direction, and closer family ties (Yellen et al, 1993). In clinical practice, late effects such as anxiety and depression are of special relevance as therapeutic interventions are available.

Before 1985, 85% of all Norwegian adult HD patients were treated at the Norwegian Radium Hospital (NRH) (Abrahamsen et al, 1993). Since 1985, NRH has treated patients from a defined region constituting half of Norway's population. Thus, a national population of HD survivors is available for a long-term follow-up study. This cross-sectional study was conducted to determine the level of psychological distress among survivors of HD and how this is related to sociodemographic variables and disease treatment characteristics as well as the predictors of anxiety and depression caseness.

### MATERIAL AND METHODS

### Subjects and data collection

Patients admitted to the NRH between 1971 and 1991 with HD and known to be alive by the end of 1993 were approached by post. Only patients who had received treatment and were between

*Received 29 November 1996*
*Revised 10 March 1997*
*Accepted 13 March 1997*

*Correspondence to:* JH Loge, Dept of Behavioural Sciences in Medicine, University of Oslo, PO Box 1111 Blindern, N-0317 Oslo, Norway

15 and 61 years at the time of diagnosis and 74 years or younger by the end of 1993 were included in this study. Non-compliers received one reminder.

### Measures

Using the Tumor Registry of NRH (Abrahamsen et al, 1993), information on date of diagnosis, stage, histology, treatment and follow-up, including current disease status, of all patients admitted to NRH for HD was available. The Norwegian version of The Hospital and Depression Scale (HADS) (Zigmond and Snaith, 1983) was included in a self-report questionnaire. The HADS consists of 14 items, measuring depression (seven items) and anxiety (seven items). Each item is scored on a four-point scale (0–3). Sum scores for either subscale (anxiety or depression respectively) are calculated by simple addition.

HADS has been used in studies of distress among cancer patients in general (Moorey et al, 1991; Carroll et al, 1993; Ibbotson et al, 1994) and among HD patients (Razavi et al, 1990). The psychometric properties are well documented (Razavi et al, 1990; Ibbotson et al, 1994) and the instrument performs well in cancer patients free of disease (Ibbotson et al, 1994). The constructors of HADS recommended two possible cut-offs (8 or higher or 11 or higher on either scale) for case definition (Zigmond and Snaith, 1983). In this study, caseness refers to the lower cut-off if not stated otherwise.

The questionnaire also included items on sociodemographic status at the time of diagnosis and at the present time. Six questions addressed psychiatric symptoms (if experienced and type of dominating symptom), psychiatric treatment (psychiatrist/psychologist, medication, hospital/clinic) and consulting a physician for psychiatric complaints. These questions were asked in relation to the time before, during and after treatment.

**Table 1** Subject characteristics ($n$ = 459)

| | $n$ (%) |
|---|---|
| Age at time of study* (yrs) | |
| ≤ 29 | 50 (11) |
| 30–39 | 117 (26) |
| 40–49 | 169 (37) |
| 50–59 | 67 (15) |
| ≥ 60 | 56 (12) |
| Gender | |
| Female | 204 (44) |
| Male | 255 (56) |
| Educational status | |
| < 10 yrs | 168 (37) |
| 11–12 yrs | 103 (23) |
| > 13 yrs | 70 (15) |
| University | 117 (25) |
| Marital status | |
| Single | 68 (15) |
| Married/cohabitant | 343 (75) |
| Divorced/separated | 35 (8) |
| Widow/widower | 13 (3) |
| Stage/substage[a] | |
| I | 119 (26) |
| II | 159 (35) |
| III | 108 (24) |
| IV | 73 (16) |
| Substage A | 312 (68) |
| Substage B | 147 (32) |
| Primary treatment | |
| Irradiation | 176 (38) |
| Chemotherapy | 79 (17) |
| Irradiation + chemotherapy | 204 (44) |
| Observational period** (yrs) | |
| 3–6 | 89 (19) |
| 7–10 | 101 (22) |
| 11–14 | 108 (24) |
| 15–23 | 161 (35) |
| Current disease status | |
| No relapse | 425 (93) |
| Relapse, currently no disease | 29 (6) |
| Relapse with current disease[b] | 5 (1) |

[a]According to the Ann Arbor staging classification; [b]relapse in 1993 or 1994. *Mean = 44, s.d. = 11.8; **mean = 12, s.d. = 5.5.

### Analysis

The data were analysed using the SPSS for Windows v. 6.1 software (SPSS, IL, USA). Statistical procedures included chi-square statistics, two-sided $t$-tests (independent samples), one-way ANOVAs, Pearson correlations, reliability estimation and multiple logistic regression. The level of significance was set at $P < 0.05$. Multiple comparisons (one-way ANOVAs) were performed by the use of Scheffé's test.

### RESULTS

A total of 557 former patients were eligible, and 459 replied (compliance rate = 82.4%). The characteristics of the respondents are given in Table 1. Mean age at time of diagnosis was 32 years (s.d. = 11.8).

There were no statistically significant differences between compliers and non-compliers in relation to age, disease stage, type of treatment or time since diagnosis. More women (86%) than men (80%) returned the questionnaire (chi-square = 3.83, $P = 0.050$).

Complete sets of either HADS anxiety or HADS depression scores were available for 439 (96%) and 447 (97%) subjects respectively. Mean anxiety score was 5.0 (s.d. = 4.1, range = 0–19). On the depression subscale, mean score was 3.1 (s.d. = 3.3, range = 0–16). The anxiety and depression subscales were strongly correlated ($r = 0.62$, $P < 0.001$). Reliability estimated by Cronbach's alpha was 0.82 and 0.86 for depression items and anxiety items respectively.

**Table 2** Anxiety and depression mean scores and cases by gender

| | Males ($n$ = 243) | Females ($n$ = 196) | $t$-value/$P$-value[a] |
|---|---|---|---|
| Anxiety (mean) | 4.6 | 5.5 | −2.2/0.028 |
| Depression (mean) | 3.3 | 3.0 | 0.95/0.34 |
| | | | Chi-square/$P$-value[b] |
| Anxiety cases (% $n$) | 19.8 | 27.6 | 3.7/0.054 |
| Depression cases (% $n$) | 13.7 | 11.6 | 0.41/0.52 |

[a]Student's $t$-tests for difference male/female mean scores with corresponding $P$-values; [b]chi-square for difference in male/female proportions with corresponding $P$-values.

© Cancer Research Campaign 1997

**Table 3** Distress in relation to educational status

| | Educational status | | | |
|---|---|---|---|---|
| | < 10 years ($n$ = 157) | 11–12 years ($n$ = 99) | > 13 years ($n$ = 70) | University ($n$ = 112) |
| Anxiety (mean) | 5.53[a] | 4.71 | 5.66 | 4.01[a] |
| Anxiety cases (% $n$) | 27[b] | 21[b] | 29[b] | 16[b] |
| Depression (mean) | 3.88[c] | 2.71[c] | 3.53 | 2.25[c] |
| Depression cases (% $n$) | 19[d] | 8[d] | 16[d] | 7[d] |

[a]$F$ = 3.61, $P$ < 0.05, by one-way ANOVA for difference in means; [b]Chi-square = 6.07, $P$ > 0.05, for difference in proportions; [c]$F$ = 6.85, $P$ < 0.05, by one-way ANOVA for difference in means; [d]Chi-square = 11.04, $P$ = 0.01, for difference in proportions.

**Table 4** Distress in relation to self-reported psychiatric symptoms before HD and during treatment

| | Psychiatric symptoms before HD | | Psychiatric symptoms during treatment | |
|---|---|---|---|---|
| | Yes ($n$ = 80) | No ($n$ = 372) | Yes ($n$ = 157) | No ($n$ = 294) |
| Anxiety (mean) | 7.6[a] | 4.5[a] | 7.0[a] | 3.8[a] |
| Anxiety cases (% $n$) | 42[b] | 19[b] | 38[b] | 14[b] |
| Depression (mean) | 4.9[a] | 2.8[a] | 4.0[a] | 2.6[a] |
| Depression cases (% n) | 31[b] | 9[b] | 19[c] | 9[c] |

[a]$P$ < 0.001 by $t$-tests for difference in means; [b]$P$ < 0.001 by chi-square for difference in proportions; [c]$P$ = 0.004 by chi-square for difference in proportions.

A total of 27% of the subjects had scores on the anxiety or the depression subscales equal to or above a caseness score of 8. A total of 4% had caseness scores on the depression subscale only and 14.5% on the anxiety subscale only, whereas 8.5% scored above 8 on both scales. If the threshold for caseness was set at 11 or higher, a total of 13% were cases. Thus, 9% were anxiety cases, 2% depression cases and 2% cases on both scales.

### Distress in relation to sociodemographic variables

The level of anxiety was higher among women than among men. Anxiety cases tended to be more frequent among women (28%) than among men (20%) ($P$ = 0.054) (Table 2).

The depression score increased with increasing age, and was higher among those aged 50–59 years (mean = 3.9, s.d. = 3.4) and 60 years or older (mean = 4.1, s.d. = 3.3) than in those under 29 years (mean = 1.8, s.d. = 1.9) ($F$ = 4.5, $P$ < 0.05). There was a linear association between increasing numbers of depression cases and increasing age (chi-square = 7.9, $P$ < 0.001), and the highest proportion of depression cases was found in the age group 50–59 years (22%).

Both the anxiety and depression scores were highest among those separated or divorced [anxiety mean = 5.9 (s.d. = 4.1), anxiety cases = 31%, depression mean = 4.2 (s.d. = 3.5), depression cases = 18%]. Their depression score was significantly higher than among the unmarried (mean = 2.1, s.d. = 2.7; $F$ = 3.68, $P$ < 0.05).

**Figure 1** Per cent anxiety (■) and depression (□) cases among subjects grouped by years since diagnosis

Subjects who had attended university had the lowest levels of both anxiety (mean = 4.1, s.d. = 3.5) and depression (mean = 2.3, s.d. = 2.8) (Table 3).

The anxiety scores differed significantly between subjects who had attended university and the poorly educated (anxiety mean = 5.5, s.d. = 4.5). The subjects educated at universities and subjects educated for 11–12 years (depression mean = 2.7, s.d. = 2.7) had lower depression scores than subjects with the least education (depression mean = 3.9, s.d. = 3.6).

The frequency of depression cases was lowest among those who had attended university (7%) and highest among subjects with the poorest education (19%) (chi-square = 11.04, $P$ = 0.01).

There were no significant differences in levels of distress or in frequency of cases in relation to habitual status at the time of diagnosis (living alone, living with their parents or cohabiting).

### Distress in relation to disease and treatment characteristics

The levels of distress or frequency of cases (both depression and anxiety) did not differ significantly in relation to stage, type of treatment (irradiation, chemotherapy or both) or number of chemotherapy treatments. However, there was a close to significant difference in the proportion of depression cases in relation to stage (stage 1, 15%; stage II, 10%; stage III, 9%; stage IV, 21%; chi-square = 7.67, $P$ = 0.053). No significant differences in levels of distress or frequency of cases were found in relation to type of irradiation (mantle vs other types), type of chemotherapy or having undergone spleenectomy or not.

Subjects with substage B had higher depressive scores (mean = 3.8, s.d. = 3.5) and more frequent depression cases (18%) than subjects with substage A (depression mean = 2.8, s.d. = 3.2; depression cases = 10%) ($t$ = –2.66, $P$ = 0.008; chi-square = 5.37, $P$ = 0.02).

Subjects observed 3–6 years after the diagnosis had the lowest depression score (mean = 2.3, s.d. = 2.4), and this was lower than in subjects observed at 7–10 years (mean = 3.8, s.d. = 3.8) ($F$ = 3.49, $P$ < 0.05) (Figure 1).

The number of both anxiety and depression cases was nearly three times higher among subjects observed 7–10 years after diagnosis than among subjects observed 3–6 years after diagnosis. This reached statistical significance only for the anxiety cases (chi-square = 8.26, $P$ = 0.04).

Relapse was not associated with any differences in levels of distress (depression mean = 2.5, s.d. = 2.7; anxiety mean = 4.3, s.d. = 3.3) or in the proportion of cases compared with subjects who had not relapsed. Of the 70 subjects (52 men, 18 women) who reported having tried but having failed to conceive children, no significant differences in either levels of distress or in the proportion of cases were found compared with those who had conceived children.

*© Cancer Research Campaign 1997* 

**Table 5** Multiple logistic regression for prediction of anxiety cases ($n$ = 425)

| Predictor variables | Number of cases (at risk) | OR[a] | 95% CI[b] |
|---|---|---|---|
| Sex | | | |
| Male | 47 (238) | – | |
| Female | 49 (187) | 1.18 | 0.70–1.98 |
| Age (continuous) | | 0.99 | 0.96–1.02 |
| Marital status | | | |
| Unmarried | 13 (61) | – | |
| Married/cohabitant | 72 (251) | 0.80 | 0.45–2.14 |
| Widow/widower | 2 (10) | 0.73 | 0.11–4.94 |
| Separated/divorced | 9 (31) | 1.02 | 0.33–3.16 |
| Educational status | | | |
| University | 17 (111) | – | |
| > 13 yrs | 19 (68) | 1.88 | 0.82–4.28 |
| 11–12 yrs | 20 (96) | 1.63 | 0.75–3.55 |
| < 10 yrs | 40 (150) | 2.07[a] | 1.02–4.22 |
| Observational period | | | |
| 3–6 yrs | 10 (82) | – | |
| 7–10 yrs | 29 (94) | 3.07[a] | 1.26–7.47 |
| 11–14 yrs | 24 (97) | 2.86[a] | 1.14–7.22 |
| 15–23 yrs | 33 (152) | 2.61[a] | 1.01–6.72 |
| Stage | | | |
| Stage I | 24 (105) | – | |
| Stage II | 34 (151) | 0.82 | 0.41–1.67 |
| Stage III | 25 (101) | 1.36 | 0.59–3.17 |
| Stage IV | 13 (68) | 1.00 | 0.36–2.79 |
| Substage | | | |
| A | 61 (288) | – | |
| B | 35 (137) | 1.03 | 0.55–1.92 |
| Primary treatment | | | |
| Chemotherapy | 11 (73) | – | |
| Chemotherapy and irradiation | 51 (195) | 2.77[a] | 1.17–6.54 |
| Irradiation | 34 (157) | 2.71 | 0.96–7.66 |
| PSP | | | |
| No | 64 (349) | – | |
| Yes | 32 (76) | 2.55[a] | 1.40–4.65 |
| PSDT | | | |
| No | 37 (272) | – | |
| Yes | 59 (153) | 3.51[a] | 2.08–5.90 |

[a]OR = odds ratio adjusted for all other variables in the table; [b]95% CI = 95% confidence intervals for odds ratio; [c] significant at 5% level; PSP, psychiatric symptoms before HD; PSDT, psychiatric symptoms during treatment.

### Distress and psychiatric symptoms before HD and during treatment

Eighty subjects (18%) stated they had experienced psychiatric symptoms before HD. Of these, 42 (53%) had been anxious, 43 (54%) had felt depressed and 18 (23%) had sleeping problems. Three had been psychotic and six had abused alcohol or drugs.

A total of 157 subjects (35%) reported psychiatric symptoms during treatment. Forty-four of these also reported psychiatric symptoms before HD. Among the former, seventy-four (48%) had been anxious, 51 (32%) had felt depressed and 47 (30%) had sleeping problems. Two had abused alcohol or drugs. Twenty-two (14%) had consulted a psychiatrist or a psychologist, and 84 (56%) had received psychotropic medication [anxiolytics, 58 (34%); hypnotics, 44 (28%)] (Table 4).

Among subjects reporting psychiatric symptoms before HD or during treatment there were higher levels of distress and more cases compared with subjects reporting no such symptoms.

### Predictors of anxiety and depression cases

Multiple logistic regression analyses were performed to identify predictors of either anxiety or depression caseness. Number and type of chemotherapy/irradiation, having relapsed or having been spleenectomized were not included as these variables were not significant predictors in the univariate analysis (Table 5).

As for anxiety cases, both the time from diagnosis (observational period 7 years or longer) and type of treatment (irradiation and chemotherapy combined) were significant predictors of caseness. Irradiation treatment solely was close to significance (odds

 © Cancer Research Campaign 1997

ratio = 2.7, 95% CI = 0.96–7.66). Low educational status, psychiatric symptoms before HD or during treatment also predicted anxiety caseness.

None of the disease or treatment variables tested in the same model predicted depression caseness. Such cases were only predicted by age (odds ratio = 1.03, 95% CI = 1.00–1.06) and psychiatric symptoms before HD (odds ratio = 5.1, 95% CI = 2.55–10.31). Similar results were repeated when anxiety and depression caseness were defined by the upper end of the borderline range (i.e. case = 11/12).

### DISCUSSION

The majority of psychosocial studies of cancer survivors have been conducted in the United States. Cultural differences and differences in treatment regimens may influence late effects, and therefore the results of the US studies cannot be generalized to the European setting (Van Tulder et al, 1994).

In the present study, the sample size, the high compliance rate, the long observational period and the standardized treatment regimens applied yield opportunities for assessing the disease and treatment variables' effects upon the psychological outcome. The study design restricted collection of data of physical late effects such as cardiopulmonary sequelae (Lund et al, 1996), which solely or in interaction with the cancer experience may affect the levels of distress (Cella and Tross, 1986; Tross and Holland, 1990). In long-term prospective studies, on the other hand, large numbers of patients are lost to follow-up.

By choosing the lower recommended threshold for case definition (Zigmond and Snaith, 1983), the proportion of false-negative cases is reduced at the possible expense of more false-positive cases. In other studies, the HADS has been employed with caseness scores ranging from 13 on both scales combined (Razavi et al, 1990), through 8 on either scale (Carroll et al, 1993) to 11 on either scale (Hopwood et al, 1991). This partly reflects the different criteria employed in validation studies of HADS, such as including adjustment disorders (Razavi et al, 1990) or omitting them (Ibbotson et al, 1994). It is therefore important to stress that HADS, like other screening instruments, gives prevalence estimates of morbidity rather than accurate measures of disorders.

Other studies of cancer survivors have found prevalences of distress cases at the same level as in the present study. As other studies have used other instruments, such comparisons are tentative. In a US study of survivors from advanced HD, the number of distress cases measured by the Brief Symptom Inventory was 22% (Kornblith et al, 1992). In another Norwegian follow-up study of cured cancer patients (head and neck cancer), 31% scored above the cut-off for caseness (measured by the General Health Questionnaire, 20-item version) (Bjordal and Kaasa, 1995). Irrespective of HADS caseness being defined by the lower threshold (e.g. 8 or higher) or the higher (e.g. 11 or higher), the prevalence of cases (mainly anxiety) in the present study is higher than in the general Norwegian population, in which the prevalence of anxiety and depression cases (measured by the Hopkins Symptoms Check List) was estimated to be 3.3% and 3.0% respectively (Moum et al, 1991). In the general population, the frequency of depression cases also increases with increasing age (Moum et al, 1991).

Compared with the general Norwegian population, anxiety cases are found relatively more frequently than depression cases. This is similar to that found among cancer patients in remission (Carroll et al, 1993) and differs from the high prevalence of depression among cancer patients with active or advanced disease (Carroll et al, 1993; McDaniel et al, 1995). Among these, the high prevalence of depression may be linked to losses, either actual such as loss of function or anticipated such as death. The high prevalence of anxiety cases after cure may therefore support the concept of a persisting stressor (Cella and Tross, 1986). Clinically, anxiety symptoms should consequently be focused in controls of cured HD patients.

### Predictors of cases

The increased number of cases 7–10 years after cure supports the concept of a non-linear relationship between distress and time since diagnosis. This may be linked to a lessening of psychological defence with time (Cella and Tross, 1986; Yellen et al, 1993). The follow-up controls at NRH, after termination of the treatment, may also have served as a 'buffer' against psychological distress. At NRH these controls have been terminated 5–10 years after cure. At termination of treatment, a 'paradoxical' rise in anxiety, specifically related to separation from staff, has been observed (Massie, 1990). Our findings may indicate a similar effect in relation to termination of controls and thereby an important psychological aspect of these controls. At termination of controls, psychological reactions to termination should therefore be addressed.

The findings of the insignificant effects of both stage and substage in relation to caseness are a reminder to medical staff that the cancer experience itself is subjective. Chemotherapeutic toxicity predicts post-treatment distress, possibly mediated through the side-effects of the more toxic regimens (Devlen et al, 1987). In the present study, those treated with the combined regimen were mostly patients with substage B and/or stage II or III. They received chemotherapy to reduce tumour masses before full irradiation regimen and thus the most extensive treatment. This supports previous findings of the treatment's impact on later psychological adjustment (Cella and Tross, 1986).

Formal educational level has been proposed as the best measure for overall socioeconomic status among cured HD patients, because the adaptional needs such as problem solving and long-term planning is affected by the educational level (Kornblith et al, 1992). Low socioeconomic status is considered to cause increased levels of distress in the general population (Wheaton, 1980). One may question whether low formal education in itself increases the risk for distress or if this finding merely reflects the general relation between social status and distress.

Time since exposure, social desirability and mode of administration affect recall (Sørensen et al, 1994). The use of psychotropics is under-reported in surveys (Sørensen et al, 1994). Recall bias may therefore affect the estimates of psychiatric problems both before and during the treatment and thus their observed predictive values on caseness. However, the number reporting psychiatric symptoms during the treatment is consistent with other prevalence estimates of psychiatric problems among lymphoma patients (Devlen et al, 1987; Razavi et al, 1992). This supports the validity of our estimates.

Detecting psychiatric problems before and during treatment is part of the clinician's duties, but such problems often remain undetected (Maguire, 1985; Cull et al, 1995). A past psychiatric history increases the risk for distress among cancer patients (Harrison and Maguire, 1994). The increased risk for anxiety caseness after cure associated with psychiatric complaints during treatment is clinically highly relevant. However, the possible recall bias and

© Cancer Research Campaign 1997

cross-sectional design of the study imply some caution regarding this finding. Nearly 50% had not received treatment (psychotropics or psychiatric counselling) during the treatment phase, but other interventions may have been applied that were undetected by our data collection. Thus, this points to possible improvements in care that might reduce the level of anxiety among the survivors.

### CONCLUSION

This study shows a rather high frequency of anxiety cases among survivors of HD. Both the increased risk associated with combined treatment and the non-linear relationship between distress and time from diagnosis are highly relevant in the follow-up programmes. The effect of distress during treatment upon psychological outcome, underlines the importance of focusing psychological disturbances in cancer care. Thus, the majority of cured HD patients survive without distress at caseness level.

### ACKNOWLEDGEMENT

This study was supported by grant no. 96028/002 from the Norwegian Cancer Society. The study was approved by The Regional Committee for Ethics in Medical Research.

### REFERENCES

Abrahamsen JF, Andersen A, Hannisdal E, Nome O, Abrahamsen AF, Kvaløy S and Høst H (1993) Second malignancies after treatment of Hodgkin's disease: the influence of treatment, follow-up time, and age. *J Clin Oncol* **11**: 255–261

Bjordal K and Kaasa S (1995) Psychological distress in head and neck cancer patients 7–11 years after curative treatment. *Br J Cancer* **71**: 592–597

Carroll BT, Kathol RG, Noyes R, Wald TG and Clamon GH (1993) Screening for depression and anxiety in cancer patients using the Hospital Anxiety and Depression Scale. *Gen Hosp Psychiatry* **15**: 69–74

Cella DF and Tross S (1986) Psychological adjustment to survival from Hodgkin's disease. *J Consult Clin Psychol* **54**: 616–622

Cull A, Stewart M and Altman DG (1995) Assessment of and intervention for psychosocial problems in routine oncology practice. *Br J Cancer* **72**: 229–235

Devlen J, Maguire P, Phillips P, Crowther D and Chambers H (1987) Psychological problems associated with diagnosis and treatment of lymphomas. *Br Med J* **295**: 953–957

Fobair P, Hoppe RT, Bloom J, Cox R, Varghese A and Spiegel D (1986) Psychosocial problems among survivors of Hodgkin's disease. *J Clin Oncol* **4**: 805–814

Harrison P and Maguire P (1994) Predictors of psychiatric morbidity in cancer patients. *Br J Psychiatry* **165**: 593–598

Hopwood P, Howell A and Maguire P (1991) Screening for psychiatric morbidity in cancer patients with advanced breast cancer: validation of two self-report questionnaires. *Br J Cancer* **64**: 353–356

Ibbotson T, Maguire P, Selby P, Priestman T and Wallace L (1994) Screening for anxiety and depression in cancer patients: the effects of disease and treatment. *Eur J Cancer* **30A**: 37–40

Koocher G and O'Malley J (1981) *The Damocles syndrome: Psychosocial Consequences of Surviving Childhood Cancer*. McGraw-Hill: New York

Kornblith AB, Anderson J, Cella DF, Tross S, Zuckerman E, Cherin E, Henderson E, Weiss RB, Cooper MR, Silver RT, Leone L, Canellos GP, Gottlieb A and Holland JC (1992) Hodgkin disease survivors at increased risk for problems in psychosocial adaption. *Cancer* **70**: 2214–2224

Lund MB, Kongerud J, Boe J, Nome O, Abrahamsen AF, Ihlen H and Forfang K (1996) Cardiopulmonary sequelae after treatment for Hodgkin's diasease: increased risk in females? *Ann Oncol* **7**: 257–264

McDaniel JS, Musselman DL, Porter MR, Reed DA and Nemeroff CB (1995) Depression in patients with cancer. *Arch Gen Psychiatry* **52**: 89–99

Maguire P (1985) Improving the detection of psychiatric problems in cancer patients. *Soc Sci Med* **20**: 819–823

Massie MJ (1990) Anxiety, panic and phobias. In *Handbook of Psychooncology*, Holland JC and Rowland JH (eds), pp. 300–309. Oxford University Press: New York

Moorey S, Greer S, Watson M, Gorman C, Rowden L, Tunmore R, Robertson B and Bliss J (1991) The factor structure and factor stability of the Hospital Anxiety and Depression Scale in patients with cancer. *Br J Psychiatry* **158**: 255–259

Moum T, Falkum E, Tambs K and Vaglum P (1991) Social background variables and mental health (in Norwegian). In *Health in Norway*, Moum T (ed.), pp. 46–62. Gyldendal: Oslo

Razavi D, Delvaux N, Farvacques C and Robaye E (1990) Screening for adjustment disorders and major depressive disorders in cancer in-patients. *Br J Psychiatry* **156**: 79–83

Razavi D, Delvaux N, Bredart A, Paesmans M, Debusscher L, Bron D and Stryckmans P (1992) Screening for psychiatric disorder in a lymphoma out-patient population. *Eur J Cancer* **28A**: 1869–1872

Sørensen HT, Møller-Petersen J, Sabroe S and Rasmussen HH (1994) Recall bias (in Danish). *Nord Med* **109**: 126–127

Tross S and Holland JC (1990) Psychological sequelae in cancer survivors. In *Handbook of Psychooncology*, Holland JC and Rowland JH (eds), pp. 101–116. Oxford University Press: New York

Van Tulder MW, Aaronsen NK and Bruning PF (1994) The quality of life of long-term survivors of Hodgkin's disease. *Ann Oncol* **5**: 153–158

Wheaton B (1980) The sociogenesis of psychological disorder: an attributional theory. *J Health Soc Behaviour* **21**: 100–124

Yellen SB, Cella DF and Bonomi A (1993) Quality of life in people with Hodgkin's disease. *Oncology* **7**: 41–45

Zigmond AS and Snaith RP (1983) The Hospital Anxiety and Depression Scale. *Acta Psychiatr Scand* **67**: 361–370

© Cancer Research Campaign 1997